# Effect of an eight-week yoga program on adolescents with Internet Gaming Disorder in an Indian school setting: a randomized controlled trial

**DOI:** 10.3389/fpubh.2026.1750580

**Published:** 2026-03-02

**Authors:** Nagalakshmi S. Rao, Raghavendra Bhat, Manoj Kumar Sharma

**Affiliations:** 1 Division of Yoga and Life Sciences, Swami Vivekananda Yoga Anusandhana Samsthana (SVYASA), Bengaluru, Karnataka, India; 2 Department of Yoga, Central University of Rajasthan, Ajmer, Rajasthan, India; 3 Department of Clinical Psychology, SHUT Clinic (Service for Healthy Use of Technology), National Institute of Mental Health & Neuro Sciences, Bengaluru, Karnataka, India

**Keywords:** adolescents, behavioral addiction, Internet Gaming Disorder, school-based intervention, yoga

## Abstract

**Background:**

Internet Gaming Disorder (IGD) is increasingly prevalent among adolescents, leading to psychological, social, and academic challenges. Yoga has demonstrated benefits in stress reduction, emotional regulation, attentional control, and overall well-being.

**Objective:**

This study aimed to evaluate the effectiveness of an eight-week Integrated Yoga Module in reducing Internet Gaming Disorder symptoms and associated psychological distress among adolescents in a school setting.

**Methods:**

In this randomized controlled trial, 120 adolescents meeting criteria for IGD were allocated to either yoga group (*n* = 60) or a control group (*n* = 60) that continued routine academic activities. Assessments were conducted at baseline and post-intervention using the Internet Gaming Disorder Scale (IGDS-20), Parental Internet Gaming Disorder Scale (PIGDS), Internet Addiction Test (IAT), Depression Anxiety Stress Scale (DASS-21), WHO Quality of Life-BREF (WHO QOL-BREF), UCLA Loneliness Scale, and Mind Wandering Questionnaire (MWQ). Data were analyzed using mixed factorial ANOVA with Group (yoga vs. control) as the between-subjects factor and Time (pre vs. post) as the within-subjects factor, with Bonferroni-adjusted *post hoc* comparisons.

**Results:**

Significant Group × Time interactions were observed across all IGDS symptom domains (salience, mood modification, tolerance, withdrawal, conflict, and relapse; all *p* < 0.001), indicating greater reductions in the yoga group relative to controls. A interaction effect was also observed for parent-reported IGD severity (*p* < 0.001). Significant interaction effects favoring the yoga group were found for internet addiction, depression, anxiety, stress, quality of life (physical, psychological, social, and environment domains), loneliness, and mind wandering (all *p* < 0.001). No significant between-group differences were observed at baseline.

**Conclusion:**

An eight-week Integrated Yoga practice demonstrated significant improvements in IGD symptoms, emotional distress, quality of life, and cognitive–emotional functioning compared with a control group. Yoga may represent a feasible and scalable complementary intervention for adolescents with IGD. Future trials incorporating active comparators and long-term follow-up are warranted.

**Clinical trial registration:**

http://ctri.nic.in/Clinicaltrials/login.php, Identifier (CTRI/2022/06/043063).

## 1 Introduction

Internet Gaming Disorder (IGD) is increasingly recognized as a behavioral addiction that predominantly affects adolescents and young adults. It is characterised by the excessive and compulsive engagement in video games- online or offline- despite negative consequences on social, academic, and emotional functioning (1, 2). IGD was first acknowledged in Section III of the *Diagnostic and Statistical Manual of Mental Disorders, (DSM-5)* as a condition warranting further research (American Psychiatric Association, 2013)*, and* it was later classified as a distinct diagnosis under the International Classification of Diseases, 11th Revision (ICD-11) by the World Health Organization (WHO) in 2018 (3, 4). This formal recognition underscores growing global concern regarding excessive gaming behaviors and their impact on adolescent mental health.

Adolescents are particularly vulnerable to IGD, given their ongoing neurobiological maturation, heightened sensitivity to rewards, and still-developing executive control mechanisms (5–7). Core symptoms of IGD include salience (preoccupation with gaming), mood modification (gaming as a coping strategy), tolerance (need for increasing gaming time), withdrawal symptoms, impaired control, conflict with family or academic responsibilities, and relapse following attempts to reduce gaming (8). These symptoms are frequently accompanied by psychological distress, including depression, anxiety, stress, loneliness, attentional difficulties, and reduced quality of life (2, 9). Neuroimaging studies further suggest that adolescents with IGD exhibit alterations in brain regions associated with reward processing, impulse control, and decision-making, resembling patterns observed in substance-related addictions (10).

Epidemiological studies suggest that Internet Gaming Disorder (IGD) affects approximately 9–10% of adolescents globally (11). However, prevalence estimates vary based on diagnostic criteria, cultural context, and assessment methodologies (12).

Despite growing awareness, there remains a paucity of empirically validated, school-based interventions targeting IGD. Cognitive Behavioral Therapy (CBT) has shown some promise, particularly in reducing impulsivity and improving emotional regulation (13–15). But its implementation in school environments is often constrained by resource limitations and the need for trained mental health professionals. Moreover, pharmacological approaches are limited and carry risks of side effects.

Yoga has emerged as a complementary mind–body practice that may offer therapeutic benefits in managing behavioral addictions (16). Rooted in Indian tradition and supported by contemporary research, yoga integrates physical postures (asanas), breathing techniques (pranayama), meditation, and lifestyle principles aimed at holistic well-being. Studies suggest that yoga can modulate the autonomic nervous system, reduce stress, enhance attention, and promote emotional regulation (17–19). These mechanisms align well with the clinical profile of IGD, which involves dysregulation in both cognitive and affective domains.

Although a few studies have examined the role of yoga in technology-related addictions, there is a lack of high-quality randomized controlled trials specifically addressing IGD among Indian adolescents (20). In this context, school settings present an ideal environment for delivering structured, low-cost, and culturally acceptable yoga-based interventions.

Hence, the current study aimed to evaluate the efficacy of an eight-week Integrated Yoga Module in mitigating symptoms of Internet Gaming Disorder (IGD) and related psychological distress among adolescents within a school environment. We posited that adolescents participating in the yoga intervention would exhibit significantly greater reductions in IGD symptoms, problematic internet use, psychological distress, loneliness, and mind wandering, as well as enhancements in quality of life, in comparison to a control group that did not receive any active intervention.

## 2 Materials and methods

### 2.1 Design of the Study

An open-label, two-arm, parallel-group randomized controlled trial was conducted from 2nd December 2022 to February 2023. This period corresponds to the mid-academic year in the Indian school calendar, following mid-term examinations and preceding final assessments.

The trial followed CONSORT guidelines for randomized controlled trials and was registered with the Clinical Trial Registry of India (CTRI/2022/06/043063) prior to participant enrolment.

### 2.2 Participants and setting

Participants were recruited from a co-educational school in Bengaluru, South India, through announcements. A total of 380 adolescents expressed interest and underwent screening. Screening was conducted using the Internet Addiction Test (IAT) to identify adolescents with problematic internet use and gaming-related behaviors. Adolescents aged 14–15 years who scored above 50 on the IAT, indicative of moderate to severe problematic internet use, were considered eligible. From the screened sample, 120 adolescents met the eligibility criteria and were enrolled in the study.

**Figure 1:**
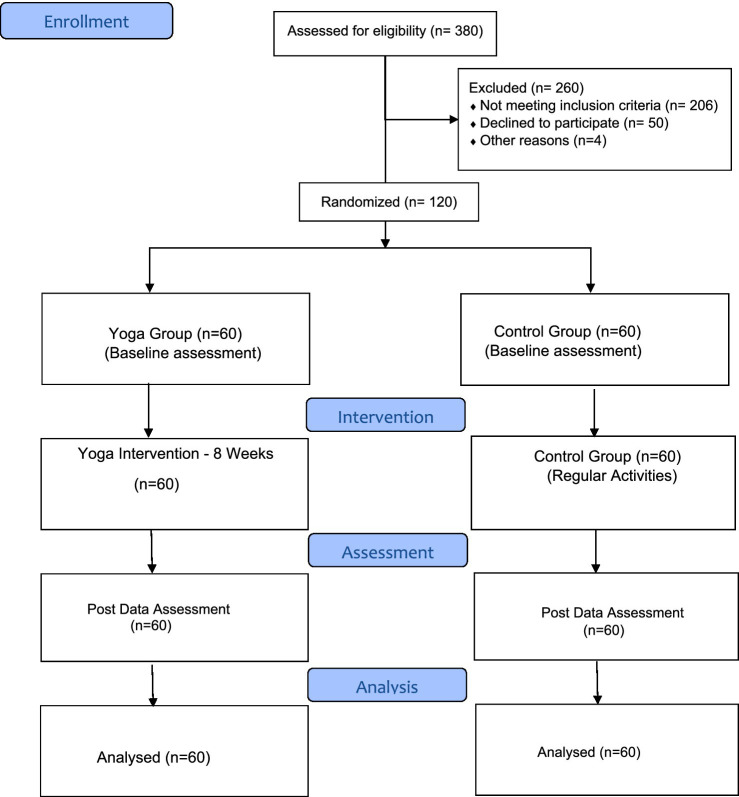
PRISMA flow diagram.

The trial profile is illustrated in the CONSORT 2010 flow diagram below (see Figure 1).

Sociodemographic and baseline characteristics are presented in Tables 1, 2, respectively.

**Table 1:** Sociodemographic characteristics of participants (*N* = 120).

Sl. No.	Characteristics (*N* = 120)	Category	*n*	Percentage (%)
1.	Gender	Male	72	60%
		Female	48	40%
2.	Class	Grade 8	120	100%
3.	Gaming time	2–3 h	104	86.6%
		3–4 h	16	13.33%

**Table 2:** Baseline characteristics of yoga group and control group.

Sl. No.	Parameters	Yoga group (*n* = 60)	Control group (*n* = 60)	*p* value
		Mean (SD)	Mean (SD)	
1.	Age in years	14.43 (0.59)	14.23 (0.43)	0.058
2.	Pre-intervention gaming time (hours/day)	2.52 (0.57)	2.50 (0.58)	0.899

Independent samples *t*-test comparing Yoga group and control group.

### 2.3 Sample size

The determination of the sample size was guided by a previously validated yoga module, as assessed in a pilot study (21). Utilizing G Power software version 3.1, the calculation was conducted with an alpha level of 0.05, a statistical power of 0.95, and an effect size of 0.839, derived from published pilot data employing the Mind Wandering Questionnaire. Although other study variables exhibited smaller effect sizes, the calculation indicated a required sample size of 38 participants. To ensure sufficient statistical power and to accommodate potential participant attrition, we recruited 60 participants for each group.

### 2.4 Randomization

Eligible participants were randomly assigned in a 1:1 ratio to either the Yoga Group or the Control Group. Randomization was performed using Research Randomizer, an online random sequence generator, by an independent individual not involved in recruitment, intervention delivery, or outcome assessment (22). Allocation concealment was ensured using sequentially numbered, opaque, sealed envelopes, which were opened only after completion of baseline assessments. Baseline equivalence between groups was examined to verify the success of randomization.

### 2.5 Inclusion and exclusion criteria

Adolescents who could communicate in English, engaged in internet games for a minimum of 2 h daily, and were willing to participate in an integrated yoga programme were included in the study. Adolescents with medical conditions, physical impairments, or substance addictions were excluded.

### 2.6 Ethical consideration

Ethical approval was obtained from the Institutional Ethics Committee of Swami Vivekananda Yoga Anusandhana Samsthana (RES/IEC–SVYASA/226/2022). Approval was obtained from school administration to conduct the study. Written informed consent was obtained from parents or legal guardians, and assent was obtained from all participating adolescents.

Participants were informed that participation was voluntary, confidentiality would be maintained, and they could withdraw at any time without academic or personal consequences.

### 2.7 Outcome measures

#### 2.7.1 Primary variable

##### 2.7.1.1 Internet Gaming Disorder Scale (IGDS)

The Internet Gaming Disorder Scale (IGDS) encompasses internet games played on a computer, laptop, gaming console, or any other device, regardless of whether they are online or offline. Twenty items require responses ranging from strongly disagree to strongly agree, focusing on gaming activity over the past year (23).

#### 2.7.2 Secondary variables

##### 2.7.2.1 Parental Internet Gaming Disorder Scale (PIGDS)

PIGDS is an adapted version of the Internet Gaming Disorder Scale (IGDS) that involves parents in assessing the same nine questions posed to the study participants.

For example, “During the last year, have you lost interest in hobbies or other activities because gaming is all you wanted to do?” In contrast, the corresponding PIGDS item inquires, “During the last year, has your child lost interest in hobbies or other activities because gaming is all she/he wanted to do?” The original IGDS uses a dichotomous response format (0 = “no,” 1 = “yes”) (24).

##### 2.7.2.2 Internet Addiction Test (IAT)

The Internet Addiction Test (IAT) is a 20-item scale that measures the presence and severity of Internet dependency among adolescents. Each statement is weighted on a Likert scale from 0 (less extreme behavior) to 5 (most extreme behavior) (25).

##### 2.7.2.3 DASS 21 (Depression and Anxiety Stress Scale)

The Depression Anxiety Stress Scales (DASS-21) consist of three self-report subscales, each featuring seven items aimed at measuring the emotional states of depression, anxiety, and stress. Respondents use a rating scale of 0, 1, 2, or 3 to indicate the frequency of applicable statements over the past week (26).

##### 2.7.2.4 WHO Quality of Life Scale (WHO QOL-BREF)

The World Health Organisation Quality of Life (WHO QOL-BREF) is an instrument that assesses four domains: physical health, psychological health, social relations, and environment, in the adolescent population, using a 5-point Likert scale. Higher scores indicate a better quality of life (27).

##### 2.7.2.5 Mind Wandering Questionnaire (MWQ)

The Mind-Wandering Questionnaire (MWQ) measures the frequency of mind wandering, intentional or spontaneous. In this study, the mind wandering scale is primarily used in adults because its simple language makes it suitable for adolescents. The questionnaire uses a 6-point Likert scale for responses: 1 (Almost never) to 6 (Almost always) (28).

##### 2.7.2.6 UCLA Loneliness Scale (University of California, Los Angeles)

The 20-item scale assesses an individual’s subjective feelings of loneliness and social isolation. Participants are required to rate each item as “O” (I often feel this way), “S” (I sometimes think this way), “R” (I rarely feel this way), or “N” (I never feel this way) (29).

### 2.8 Intervention

The intervention was based on a validated Integrated Yoga Module developed specifically to address Internet Gaming Disorder (IGD) in adolescents (21). The module was conceptualized using the Pancha Kosha framework from the Taittiriya Upanishad, which describes human existence at five interrelated levels—Annamaya (physical), Pranamaya (energy), Manomaya (emotional), Vijnanamaya (intellectual), and Anandamaya (bliss). Practices were selected to target each sheath, with the understanding that interventions at one level influence the others. The design of the module was also informed by guidance from the *Bhagavad Gita* (Chapter 2, Verses 62–63) on cultivating detachment and moderation to overcome excessive attachment behaviors such as IGD.

The module content was finalized through a structured group discussion with five yoga experts and one psychiatrist experienced in treating IGD. An initial practice list of 34 items, derived from classical yoga texts (*Hatha Yoga Pradipika*, *Gheranda Samhita*, *Yoga Vasishta*, *Patanjali Yoga Sutras*, and others) and contemporary scientific literature, was reviewed for relevance and therapeutic potential. The final validated module comprised: loosening exercises, asanas (postures), Pranayama (breathing techniques), mindfulness meditation, Surya Namaskara (sun salutations), deep relaxation technique.

The eight-week yoga program was delivered to the intervention group by an experienced yoga therapist, with sessions held 3 days per week, each lasting 40 min. Sessions were conducted in the school premises during regular academic hours. The details of the yoga module is presented in Table 3.

**Table 3:** Validated yoga module for IGD for 40 min session.

Sl. No.	List of the practices	Number of rounds	Duration
1	Opening prayer		1 min
Loosening practices			
2	Forward & backward bending	5 rounds	1 min
3	Bend and twist (alternate toe touching)	5 rounds	1 min
4	Jogging	5 rounds	1 min
5	Mani Bhanda Shakthi Vikashaka Vyayama (wrist movement)	5 rounds	1 min
6	Anguli Shakthi Vikashaka Vyayama (finger movement)	5 rounds	1 min
7	Kara Tala Shakthi Vikashaka Vyayama (palm movement)	5 rounds	1 min
8	Kara Pishta Shakthi Vikashaka Vyayama (back of the hand movement)	5 rounds	1 min
9	Khaponi Vikashaka Shakthi Vyayama (elbow movement)	5 rounds	1 min
10	Bhuja Bhanda Shakthi Vyayama (shoulder movement)	5 rounds	1 min
11	Griva Shakthi Vikashaka Vyayama (neck movement)	5 rounds	1 min
12	Netra Shakthi Vikashaka Vyayama (eye movement)	5 rounds	1 min
13	Suryanamaskara (sun salutation)	6 rounds	3 min
Breathing practices			
14	Vyagrah Shvasa (tiger breathing)	3rounds	1 min
15	Shashanka Shvasa (rabbit breathing)	3rounds	1 min
Standing postures			
16	Parivritha Trikonasana (revolved triangle posture)	1 round	1 min
17	Vrukshasana (tree posture)	1 round	1 min
18	Veerabhadrasana (warrior posture)	1 round	1 min
Sitting postures			
19	Paschimothanasana (seated forward bend)	1 round	1 min
20	Ushtrasana (camel posture)	1 round	1 min
Supine postures			
21	Poornanavasana (full boat posture)	1 round	1 min
22	Setubandasana (bridge posture)	1 round	1 min
23	Uttanapadasana (raised leg posture)	1 round	1 min
Prone postures			
24	Bhujangasana (cobra posture)	1 round	1 min
25	Dhanurasana (bow posture)	1 round	1 min
26	Balasana (baby posture)	1 round	1 min
27	Pranayama (regulated breathing practice)		
	Nadishuddhi (alternate nostril breathing)	5 rounds	2 min
	Bhramari (humming of bumblebee breathing)	5 rounds	2 min
28	Deep relaxation technique (DRT)	–	5 min
29	Dhyana (mindfulness meditation)	–	2 min
30	Closing prayer		1 min

Participants in the control group did not receive any yoga-based intervention and continued with their usual academic activities.

### 2.9 Data analysis

Data were analyzed using mixed factorial analysis of variance (ANOVA) to examine intervention effects over time. For each outcome variable, Group (yoga vs. control) was specified as the between-subjects factor, and Time (pre-intervention and post-intervention) was specified as the within-subjects factor. The primary effect of interest was the Group × Time interaction, which tests whether changes over time differed between the intervention and control groups and is considered the most appropriate inferential statistic for randomized controlled pre–post designs. Prior to analysis, data distributions were examined for normality using the Kolmogorov–Smirnov test and Q–Q plot. Given the robustness of mixed ANOVA to moderate deviations from normality and the balanced design with equal time points, parametric analyses were retained. Where significant interaction effects were observed, Bonferroni-adjusted *post hoc* comparisons of estimated marginal means were conducted to control for multiple testing. Effect sizes were reported as partial eta squared (*η*^2^*p*). Statistical analyses were performed using Jamovi (version 2.7), with the level of significance set at *p* < 0.05.

## 3 Results

### 3.1 Sociodemographic and baseline characteristics

Of the 380 adolescents screened, 120 met the eligibility criteria and were enrolled in the study. All participants were in Grade 8. Among them, 60% were male (*n* = 72) and 40% were female (*n* = 48). The majority were 14 years old (64.16%), and the remaining 35.83% were 15 years old. Regarding gaming behavior, 86.67% reported playing internet games for 2–3 h per day, while 13.33% played for 3–4 h. No significant differences were observed between the yoga and control groups in terms of baseline sociodemographic variables. Data from all 120 participants were included in the final analysis, as there were no dropouts. Sociodemographic and baseline characteristics are presented in Tables 1, 2, respectively.

### 3.2 Mixed factorial analyses of variance (ANOVA)

Outcome variables were analyzed using mixed factorial analyses of variance (ANOVA) with Group (yoga vs. control) as the between-subjects factor and Time (pre-intervention vs. post-intervention) as the within-subjects factor. Intervention effects were evaluated primarily using the Group × Time interaction term. *Post hoc* comparisons of estimated marginal means were conducted using Bonferroni correction to control for multiple comparisons. Mixed factorial ANOVA results are presented in Table 4, and Bonferroni-adjusted *post hoc* comparisons are presented in Table 5. Baseline comparisons confirmed no significant differences between groups across all outcome variables (all *p* > 0.05). Group mean values ± Standard Deviation for all the outcome variables for yoga and control group presented in Table 6.

**Table 4:** Summary of mixed factorial ANOVA results.

Variable	Factor	*F*	df	*P* (level of significance)	*η*^2^*p* (effect size)
Internet Gaming Disorder Scale (IGDS)					
Salience factor	Time	449.55	1, 118	<0.001	0.79
	Group	133.47	1, 118	<0.001	0.53
	Time × Group	89.33	1, 118	<0.001	0.43
Mood modification factor	Time	397.60	1, 118	<0.001	0.77
	Group	147.70	1, 118	<0.001	0.56
	Time × Group	88.53	1, 118	<0.001	0.43
Tolerance factor	Time	566.58	1, 118	<0.001	0.83
	Group	101.75	1, 118	<0.001	0.46
	Time × Group	221.29	1, 118	<0.001	0.65
Withdrawal symptoms factor	Time	384.68	1, 118	<0.001	0.77
	Group	89.24	1, 118	<0.001	0.43
	Time × Group	50.06	1, 118	<0.001	0.30
Conflict factor	Time	29.44	1, 118	<0.001	0.20
	Group	74.18	1, 118	<0.001	0.39
	Time × Group	50.34	1, 118	<0.001	0.30
Relapse factor	Time	190.23	1, 118	<0.001	0.62
	Group	59.91	1, 118	<0.001	0.34
	Time × Group	71.54	1, 118	<0.001	0.38
Parental Internet Gaming Disorder Scale (PIGDS)	Time	523.52	1, 118	<0.001	0.82
	Group	86.34	1, 118	<0.001	0.42
	Time × Group	395.31	1, 118	<0.001	0.77
Internet Addiction Test	Time	79.64	1, 118	<0.001	0.40
	Group	73.21	1, 118	<0.001	0.38
	Time × Group	113.67	1, 118	<0.001	0.49
DASS21					
Depression	Time	176.65	1, 118	<0.001	0.60
	Group	149.89	1, 118	<0.001	0.56
	Time × Group	149.34	1, 118	<0.001	0.56
Anxiety	Time	112.35	1, 118	<0.001	0.49
	Group	129.21	1, 118	<0.001	0.52
	Time × Group	112.35	1, 118	<0.001	0.49
Stress	Time	140.44	1, 118	<0.001	0.54
	Group	78.42	1, 118	<0.001	0.40
	Time × Group	140.44	1, 118	<0.001	0.54
WHO BREF QOL					
Physical health	Time	3.47	1, 118	0.065	0.03
	Group	24.33	1, 118	<0.001	0.17
	Time × Group	22.40	1, 118	<0.001	0.16
Psychological health	Time	16.69	1, 118	<0.001	0.12
	Group	47.34	1, 118	<0.001	0.29
	Time × Group	48.94	1, 118	<0.001	0.29
Social relationships	Time	199.57	1, 118	<0.001	0.63
	Group	17.06	1, 118	<0.001	0.13
	Time × Group	47.14	1, 118	<0.001	0.29
Environment	Time	161.14	1, 118	<0.001	0.58
	Group	60.10	1, 118	<0.001	0.34
	Time × Group	59.03	1, 118	<0.001	0.33
UCLA Loneliness Scale	Time	160.35	1, 118	<0.001	0.58
	Group	157.07	1, 118	<0.001	0.57
	Time × Group	196.87	1, 118	<0.001	0.63
Mind wandering	Time	71.87	1, 118	<0.001	0.38
	Group	71.92	1, 118	<0.001	0.38
	Time × Group	124.10	1, 118	<0.001	0.51

**Table 5:** *Post hoc* multiple comparisons (between group and with-in group) with Bonferroni correction.

Variable	Comparison	Mean difference	*SE*	*p* (adjusted)
Internet Gaming Disorder Scale (IGDS)				
Salience factor	Yoga vs. Control (Pre)	0.21	0.09	0.152
	Yoga vs. Control (Post)	1.38	0.09	<0.001
	Yoga (Pre − Post)	1.90	0.09	<0.001
	Control (Pre − Post)	0.73	0.09	<0.001
Mood modification factor	Yoga vs. Control (Pre)	0.21	0.09	0.099
	Yoga vs. Control (Post)	1.37	0.09	<0.001
	Yoga (Pre − Post)	1.81	0.09	<0.001
	Control (Pre − Post)	0.65	0.09	<0.001
Tolerance factor	Yoga vs. Control (Pre)	0.11	0.07	0.645
	Yoga vs. Control (Post)	1.29	0.08	<0.001
	Yoga (Pre − Post)	1.83	0.07	<0.001
	Control (Pre − Post)	0.42	0.07	<0.001
Withdrawal symptoms factor	Yoga vs. Control (Pre)	0.11	0.11	1.000
	Yoga vs. Control (Post)	1.20	0.10	<0.001
	Yoga (Pre − Post)	2.05	0.11	<0.001
	Control (Pre − Post)	0.96	0.11	<0.001
Conflict factor	Yoga vs. Control (Pre)	0.07	0.09	1.000
	Yoga vs. Control (Post)	0.92	0.07	<0.001
	Yoga (Pre − Post)	0.75	0.08	<0.001
	Control (Pre − Post)	0.10	0.08	1.000
Relapse factor	Yoga vs. Control (Pre)	0.12	0.12	1.000
	Yoga vs. Control (Post)	1.21	0.09	<0.001
	Yoga (Pre − Post)	1.74	0.11	<0.001
	Control (Pre − Post)	0.42	0.11	0.002
Parental Internet Gaming Disorder Scale (PIGDS)	Yoga vs. Control (Pre)	0.17	0.27	1.000
	Yoga vs. Control (Post)	4.37	0.261	<0.001
	Yoga (Pre − Post)	4.52	0.15	<0.001
	Control (Pre − Post)	0.32	0.15	0.217
Internet Addiction Test	Yoga vs. Control (Pre)	0.01	0.10	1.000
	Yoga vs. Control (Post)	1.34	0.10	<0.001
	Yoga (Pre − Post)	1.24	0.09	<0.001
	Control (Pre − Post)	0.11	0.09	1.000
DASS21				
Depression	Yoga vs. Control (Pre)	0.23	0.20	1.000
	Yoga vs. Control (Post)	4.80	0.34	<0.001
	Yoga (Pre − Post)	4.77	0.26	<0.001
	Control (Pre − Post)	0.20	0.26	1.000
Anxiety	Yoga vs. Control (Pre)	0.03	0.03	1.000
	Yoga vs. Control (Post)	2.90	0.26	<0.001
	Yoga (Pre − Post)	2.87	0.19	<0.001
	Control (Pre − Post)	0.00	0.19	1.000
Stress	Yoga vs. Control (Pre)	0.23	0.24	1.000
	Yoga vs. Control (Post)	4.57	0.39	<0.001
	Yoga (Pre − Post)	4.33	0.26	<0.001
	Control (Pre − Post)	0.00	0.26	1.000
WHO QOL BREF				
Physical health	Yoga vs. Control (Pre)	4.57	2.87	0.686
	Yoga vs. Control (Post)	18.58	2.68	<0.001
	Yoga (Pre − Post)	9.77	2.09	<0.001
	Control (Pre − Post)	4.25	2.09	0.268
Psychological health	Yoga vs. Control (Pre)	1.02	2.21	1.000
	Yoga vs. Control (Post)	27.67	3.32	<0.001
	Yoga (Pre − Post)	22.72	2.90	<0.001
	Control (Pre − Post)	5.97	2.90	0.251
Social relationships	Yoga vs. Control (Pre)	2.43	2.68	1.000
	Yoga vs. Control (Post)	24.17	3.77	<0.001
	Yoga (Pre − Post)	40.67	2.74	<0.001
	Control (Pre − Post)	14.07	2.74	<0.001
Environment	Yoga vs. Control (Pre)	1.98	2.31	1.000
	Yoga vs. Control (Post)	31.70	3.41	<0.001
	Yoga (Pre − Post)	44.67	3.10	<0.001
	Control (Pre − Post)	10.98	3.10	0.003
UCLA Loneliness Scale	Yoga vs. Control (Pre)	0.03	0.69	1.000
	Yoga vs. Control (Post)	15.42	0.94	<0.001
	Yoga (Pre − Post)	14.63	0.78	<0.001
	Control (Pre − Post)	0.75	0.78	1.000
Mind wandering	Yoga vs. Control (Pre)	0.27	0.20	1.000
	Yoga vs. Control (Post)	2.38	0.14	<0.001
	Yoga (Pre − Post)	2.33	0.17	<0.001
	Control (Pre − Post)	0.32	0.17	0.373

**Table 6:** Group mean values ± standard deviation for all the outcome variables for yoga and control group.

Variable	Yoga		Control	
	Mean (SD)		Mean (SD)	
	Pre	Post	Pre	Post
IGD				
Salience	3.49 (0.360)	1.59 (0.565)	3.70 (0.605)	2.97 (0.458)
Mood modification	3.49(0.297)	1.68(0.553)	3.70(0.602)	3.05(0.458)
Tolerance	3.44(0.304)	1.61(0.504)	3.33(0.445)	2.91(0.369)
Withdrawal symptoms	3.52 (0.606)	1.47 (0.566)	3.63 (0.612)	2.67 (0.475)
Conflict	2.83 (0.557)	2.08 (0.447)	2.90 (0.440)	3.00 (0.343)
Relapse	3.50 (0.648)	1.76 (0.517)	3.38 (0.663)	2.96 (0.447)
PIGDS	7.08 (1.266)	2.57 (0.871)	7.25 (1.70)	6.93 (1.79)
Internet Addiction Test	53.65 (12.41)	24.05 (7.597)	50.45 (11.72)	52.73 (11.22)
DASS21				
Depression	9.23 (1.226)	4.47(2.310)	9.47(0.965)	9.27(1.219)
Anxiety	5.97 (0.258)	3.10 (1.997)	6.00 (0.000)	6.00 (0.368)
Stress	9.57 (1.047)	5.23 (2.632)	9.80 (1.549)	9.80 (1.549)
WHO BREF QOL				
Physical health	46.8 (17.88)	56.6 (13.02)	42.3 (13.2)	38.0 (16.1)
Psychological health	40.2 (8.92)	62.9 (18.68)	41.2 (14.6)	35.3 (17.7)
Social relationships	34.0 (11.00)	74.7 (16.08)	36.5 (17.6)	50.5 (24.3)
Environment	38.2 (10.62)	82.8 (15.42)	40.2 (14.4)	51.1 (21.5)
UCLA Loneliness Scale	45.8 (3.39)	31.1 (4.54)	45.8 (4.12)	46.6 (5.71)
Mind wandering	3.75 (1.174)	1.42 (0.530)	3.48 (1.017)	3.80 (0.935)

#### 3.2.1 Internet Gaming Disorder Scale (IGDS-20)

A significant Group × Time interaction was observed for all IGDS symptom domains, including salience [*F* (1, 118) = 89.33, *p* < 0.001, *η*^2^*p* = 0.43], mood modification [*F* (1, 118) = 88.53, *p* < 0.001, *η*^2^*p* = 0.43], tolerance [*F* (1, 118) = 221.29, *p* < 0.001, *η*^2^*p* = 0.65], withdrawal [*F* (1, 118) = 50.06, *p* < 0.001, *η*^2^*p* = 0.30], conflict [*F*(1, 118) = 50.34, *p* < 0.001, *η*^2^*p* = 0.30], and relapse [*F*(1, 118) = 71.54, *p* < 0.001, *η*^2^*p* = 0.38], indicating a significantly greater reduction in the yoga group compared with the control group over time.

Bonferroni-adjusted *post hoc* analyses showed significantly lower post-intervention scores in the yoga group compared with the control group across all domains of Internet Gaming Disorder Scale.

#### 3.2.2 Parental Internet Gaming Disorder Scale (PIGDS)

A significant Group × Time interaction was observed for Parental Internet Gaming Disorder Scale [*F* (1, 118) = 395.31, *p* < 0.001, *η*^2^*p* = 0.77], indicating a significantly greater reduction in IGD symptoms in the yoga group compared with the control group over time.

Bonferroni-adjusted *post hoc* analyses further demonstrated that post-intervention parent-reported IGD scores were significantly lower in the yoga group than in the control group.

#### 3.2.3 Internet Addiction Test

A significant Group × Time interaction was observed for internet addiction severity, as assessed by the Internet Addiction Test [*F* (1, 118) = 113.67, *p* < 0.001, *η*^2^*p* = 0.49], indicating a significantly greater reduction in internet addiction symptoms in the yoga group compared with the control group over time.

Bonferroni-adjusted *post hoc* analyses further demonstrated that post-intervention IAT scores were significantly lower in the yoga group than in the control group.

#### 3.2.4 Depression Anxiety Stress Scale (DASS-21)

Significant Group × Time interactions were observed for all DASS-21 domains, including depression [*F* (1, 118) = 149.34, *p* < 0.001, *η*^2^*p* = 0.56], anxiety [*F* (1, 118) = 112.35, *p* < 0.001, *η*^2^*p* = 0.49], and stress [*F* (1, 118) = 140.44, *p* < 0.001, *η*^2^*p* = 0.54], indicating significantly greater reductions in negative emotional states in the yoga group compared with the control group over time. Bonferroni-adjusted post hoc analyses further demonstrated that post-intervention depression, anxiety, and stress scores were significantly lower in the yoga group than in the control group.

#### 3.2.5 WHO Quality of Life (QOL-BREF)

Significant Group × Time interactions were observed across all domains of the WHO QOL-BREF, including physical health [*F* (1, 118) = 22.40, *p* < 0.001, *η*^2^*p* = 0.16], psychological health [*F* (1, 118) = 48.94, *p* < 0.001, *η*^2^*p* = 0.29], social relationships [*F* (1, 118) = 47.14, *p* < 0.001, *η*^2^*p* = 0.29], and environment [*F* (1, 118) = 59.03, *p* < 0.001, *η*^2^*p* = 0.33]. These interaction effects indicate significantly greater improvements in quality of life in the yoga group compared with the control group over time. Bonferroni-adjusted *post hoc* analyses further demonstrated that post-intervention WHO QOL-BREF domain scores were significantly higher in the yoga group than in the control group.

#### 3.2.6 UCLA loneliness scale

A significant Group × Time interaction was observed for loneliness [*F* (1, 118) = 196.87, *p* < 0.001, *η*^2^*p* = 0.63], indicating a significantly greater reduction in loneliness levels in the yoga group compared with the control group over time. Bonferroni-adjusted *post hoc* analyses further demonstrated that post-intervention loneliness scores were significantly lower in the yoga group than in the control group.

#### 3.2.7 Mind wandering

A significant Group × Time interaction was observed for mind wandering [*F* (1, 118) = 124.10, *p* < 0.001, *η*^2^*p* = 0.51], indicating a significantly greater reduction in mind-wandering tendencies in the yoga group compared with the control group over time. Bonferroni-adjusted post hoc analyses further demonstrated that post-intervention MWQ scores were significantly lower in the yoga group than in the control group.

## 4 Discussion

The present randomized controlled trial examined the effectiveness of an eight-week Integrated Yoga Module for adolescents with Internet Gaming Disorder (IGD) in a school setting. The findings demonstrated that adolescents who participated in the yoga intervention exhibited significantly greater reductions over time, relative to the control group, across all core IGD symptom domains, including salience, mood modification, tolerance, withdrawal, conflict, and relapse. In addition, significant intervention-related effects were observed for parent-reported IGD severity, internet addiction, depression, anxiety, and stress, all domains of quality of life, loneliness, and mind-wandering. The convergence of self-reported and parent-reported outcomes strengthens confidence in the behavioral changes associated with the intervention and reduces concerns related to mono-informant bias.

The most prominent findings of the study were the consistent Group × Time interaction effects observed across all IGD symptom domains assessed using the IGDS-20, as well as parent-reported IGD severity measured by the PIGDS. This pattern suggests that the Integrated Yoga Module was associated with broad-based reductions in core features of IGD rather than isolated symptom changes. These findings are consistent with emerging evidence indicating that mind–body interventions, including yoga and mindfulness-based practices, may be effective in reducing maladaptive technology use and enhancing self-regulatory capacities among adolescents (30, 31).

The significant interaction observed for internet addiction severity further suggests that the benefits of the Integrated Yoga Module may extend beyond gaming-specific behaviors to more generalized patterns of problematic internet use. This is particularly relevant given the substantial conceptual and behavioral overlap between Internet Gaming Disorder and generalized internet addiction in adolescent populations.

In addition to behavioral outcomes, significant Group × Time interaction effects were observed for depression, anxiety, and stress, indicating that the yoga intervention was associated with greater reductions in negative emotional states compared with the control condition. These findings align with prior research demonstrating the efficacy of yoga-based practices in improving emotional regulation, stress tolerance, and affective balance among adolescents and young adults (14).

Recent evidence from athletic populations provides a useful theoretical framework for interpreting these findings. Solmaz and Yarayan (32) demonstrated that mindfulness moderates the relationship between threat appraisal and negative emotional states, highlighting its buffering role in stress-related affective responses. Given that the Integrated Yoga Module incorporated mindfulness, breath awareness, and attentional regulation practices, similar mechanisms may underlie the observed reductions in anxiety, stress, and mind wandering among adolescents with IGD. Enhanced present-moment awareness and reduced cognitive reactivity may facilitate disengagement from maladaptive gaming-related coping strategies.

The present study also demonstrated significant interaction effects across all domains of quality of life, including physical health, psychological health, social relationships, and environmental well-being. These findings suggest that the benefits of yoga extend beyond symptom reduction to broader improvements in daily functioning and subjective well-being. Similarly, the observed reduction in loneliness highlights the potential role of structured, group-based yoga practice in addressing social isolation, a common concern among adolescents with excessive gaming behaviors.

In this context, findings reported by Solmaz (33) offer additional insight, demonstrating that psychosocial resources such as self-esteem and perceived social support can mitigate the negative impact of dispositional vulnerabilities on subjective well-being in young athletes. Although these constructs were not directly assessed in the present study, improvements in quality of life and reductions in loneliness may reflect similar protective psychosocial mechanisms fostered through regular group engagement, shared practice, and supportive instructor–participant interactions inherent in the yoga intervention.

Cognitive-behavioral therapy (CBT) is currently regarded as one of the most empirically supported psychological interventions for Internet Gaming Disorder, with evidence supporting its effectiveness in reducing gaming behaviors, maladaptive cognitions, and associated psychological distress (15). CBT-based approaches primarily target dysfunctional gaming-related beliefs, impaired impulse control, and maladaptive coping strategies through cognitive restructuring and behavioral modification techniques (34).

While the present study did not include CBT as an active comparator, the observed reductions across core IGD symptom domains suggest that the Integrated Yoga Module may influence overlapping but distinct therapeutic pathways. Yoga-based interventions emphasize self-regulation through attentional control, emotional awareness, stress reduction, and autonomic balance, which may indirectly reduce gaming behaviors by enhancing emotional stability and adaptive coping. In contrast to CBT, which is typically delivered by trained mental health professionals, yoga interventions may be more feasible and scalable within school settings, particularly in resource-limited contexts.

Importantly, the present findings should not be interpreted as indicating superiority of yoga over CBT. Rather, the results suggest that yoga may represent a complementary or alternative intervention, especially for adolescents with limited access to psychological services or those who prefer non-stigmatizing, group-based approaches.

### 4.1 Strengths and limitations

This study’s strengths include its randomized controlled design, school-based implementation, high adherence, absence of attrition, and use of both self-reported and parent-reported outcome measures. Nevertheless, several limitations should be acknowledged. The open-label design and reliance on self-report measures may introduce expectancy effects, social desirability bias, and demand characteristics, potentially inflating observed effects. The use of a passive control group limits the ability to isolate yoga-specific effects from non-specific factors such as social interaction, structured activity, or time away from gaming. Additionally, the single-school urban sample may limit generalizability, and the absence of long-term follow-up precludes conclusions about the sustainability of benefits.

### 4.2 Implications and future directions

Despite these limitations, the findings suggest that yoga may be a feasible, culturally acceptable, and cost-effective school-based intervention for adolescents with IGD. Future research should employ active or attention-matched control conditions, incorporate objective behavioral measures (e.g., digital usage tracking), and include physiological markers such as heart rate variability or cortisol to further elucidate underlying mechanisms. Longitudinal studies are also needed to assess the durability of behavioral change and the role of yoga in preventing relapse. Comparative trials examining yoga alongside established interventions such as CBT or structured physical activity would further clarify its relative and complementary benefits.

## 5 Conclusion

This randomized controlled trial demonstrates that an eight-week Integrated Yoga Module delivered in a school setting was associated with significantly greater improvements over time in Internet Gaming Disorder symptoms, parent-reported IGD severity, internet addiction, emotional distress, quality of life, loneliness, and mind wandering compared with a control condition. The consistency between self-reported and parent-reported outcomes strengthens confidence in the observed effects. While not a substitute for established psychological treatments such as cognitive-behavioral therapy, yoga may represent a feasible and scalable complementary intervention for adolescents with Internet Gaming Disorder, particularly within school-based and resource-limited settings. Further studies with active comparators and long-term follow-up are warranted.

## Data Availability

The raw data supporting the conclusions of this article will be made available by the authors, without undue reservation.
